# A simple prototype for assessing plant cold hardiness with differential thermal analysis

**DOI:** 10.1016/j.ohx.2026.e00749

**Published:** 2026-02-03

**Authors:** John R. Butnor

**Affiliations:** USDA Forest Service, Northern Research Station, Burlington, VT, USA

**Keywords:** Cold tolerance, Cold hardiness, Thermoelectric module, TEM, Differential thermal analysis, DTA

## Abstract

Differential thermal analysis (DTA) is a laboratory technique used to assess cold tolerance of plant tissue by detecting low temperature exotherms released when intracellular water freezes and cells are damaged. Measurements are made by placing samples on thermoelectric modules (TEMs) in a programmable freezer, slowly reducing the temperature, and detecting the latent heat of fusion released when intracellular water undergoes a phase change from liquid to solid. TEMs are solid-state Peltier devices that are commonly used for heating or cooling of electronics depending on the direction of the applied electrical current. In their quiescent state, they are very effective heat flux sensors that can be used to detect minute changes in temperature. Configurations that multiplex many TEMs for DTA have been used for several decades to assess cold tolerance, but the details of how they are constructed and programmed are limited. Here I present a simple prototype for assessing plant cold hardiness with DTA using a legacy datalogger and TEMs as a proof of concept. By using single-ended voltage measurements to monitor the TEMs, the number of samples which can be monitored can be maximized, thereby reducing the need for multiplexers. This prototype demonstrates that DTA can be accessible to researchers using basic dataloggers.

## Graphical abstract

**Figure:**
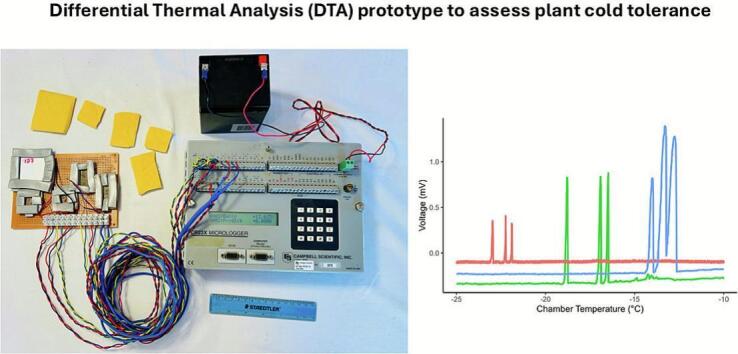

Specifications table

**Table:** 

Hardware name	*Differential thermal analysis prototype*
Subject area	• Environmental, planetary and agricultural sciences
Hardware type	• Measuring physical properties using in-lab sensors
Closest commercial analog	While no commercial analog is available, Brock University Technical Services, Electronics Shops, Guelph, Canada, has fabricated multiplexed thermoelectric module systems and software for researchers studying plant cold tolerance under laboratory conditions in the past.
Open source license	This work was performed by US government employees on official time and is therefore in the public domain: Creative Commons Zero (CC0) license is applied.
Cost of hardware	Cost with legacy Campbell Scientific datalogger ∼$400 USD. Cost with purchase of Campbell Scientific data CR1000x logger and control software ∼$2,400 USD. Access to a programmable temperature chamber is also required for operation.
Source file repository	Control code, interpretation R code, interpretation HTML code, and biological case example data available here: https://doi.org/10.5281/zenodo.17228047

## 1 Hardware in context

Differential thermal analysis (DTA) is a research protocol for quantifying plant tissue cold tolerance under laboratory conditions. It takes advantage of physical properties of water where a phase change from a supercooled liquid to solid results in a release of detectable heat. Plant tissue samples such as shoots, roots, leaves or dormant buds are placed in a programmable freezer where the air temperature is slowly lowered (∼2–6 °C per hour) and a sensor system detects the release of heat generated by the phase change. These sensors could be traditional thermal sensors such as thermocouples and thermistors or thermoelectric modules (TEMs) which make multiple measurements per minute to detect a change in temperature [1], [2]. Thermocouples and thermistors can be problematic in several ways, such as needing to be simultaneously in direct contact with the plant tissue and sensitive enough to detect minute temperature changes. TEMs are commonly used in electronics as thermoelectric heat pumps capable of heating or cooling components by the transfer of heat to the opposite side of the module. The module is constructed of two dissimilar conductors fixed together and utilizes the “Peltier effect” wherein a DC current applied in one direction, cools the module on one side and heats the other side. When the current is reversed, the opposite thermal effect is observed [3]. In their quiescent state without any application of external current, TEMs can be very effective heat flux sensors [4] and have been adopted as the current state-of-the-art technology in DTA analysis. While they cannot directly measure temperature, small changes in temperature between the two sides of the module create a detectable millivolt signal that may be recorded by a general purpose datalogger. Current embodiments of DTA systems involve arranging TEMs on trays, creating wells or compartments around each TEM to hold plant tissue in place, having a reference thermistor or thermocouple to measure the ambient chamber temperature, and connecting each TEM to a logging voltmeter or any modern datalogger [1], [2]. While no commercially available DTA system currently exists, Brock University Technical Services, Electronics Shops, in Guelph, Canada, has fabricated multiplexed thermoelectric module systems and software for researchers studying plant cold tolerance under laboratory conditions in the past.

My purpose in creating the simple DTA prototype is to highlight just how basic the design and programming of such a system can be. This work serves to demystify the perceived complexities of DTA systems and heat flux sensing applications using TEMs to make the approach more accessible. There are numerous studies that detail improvements to DTA exotherm detection equipment [5], important issues surrounding tissue preparation and handling [6], as well as insightful treatments of DTA data interpretation [7], [8]. This simple DTA prototype project does not discount the incremental gains made over decades of research on plant cold tolerance quantification. Rather, it simplifies the technological aspects of DTA systems, reduces overall costs by using commonly available dataloggers and supplies, and increases sample capacity with a multi-TEM configuration thereby enabling researchers to more easily participate in plant cold hardiness studies using DIY instrumentation. As the title indicates, this is a *simple prototype* to give the reader the tools to design a configuration and make modifications that suit the needs of their specific DTA application, perhaps in ways that I wouldn’t have guessed *a priori*. It is important to note that DTA systems require a programmable temperature chamber to gradually expose plant tissue to temperatures ranging from + 10 °C to −45 °C. This is a significant cost outside of the DTA prototype, but this type of equipment may be available as a shared resource at universities or agricultural laboratories.

## 2 Hardware description

Multiplexed TEM systems for DTA assessment have been developed by various researchers and institutions, but simple designs are not readily available. Existing designs use relatively large TEMs (39.9 mm × 39.9 mm) arranged on trays that can only accommodate 9 or 10 samples [1]. These TEMs are appropriate for larger plant tissue types, such as grapevine buds and canes, or multiple dormant buds in composite samples. However, the use of smaller micro-TEMs make it possible to incorporate more TEMs per tray with the added benefit of enhanced sensitivity and exotherm detection from smaller plant tissue, such as dormant buds of North American forest trees. Following discussions with representatives of TE Technology, Inc., Traverse City, Michigan, USA, regarding TEM selection, I selected two micro TEMs measuring 12 mm × 13 mm and 12 mm × 26 mm as well as one standard TEM measuring 39.9 mm × 39.9 mm. TEMs are available with the option to environmentally ruggedize them through a process called “potting” where the air spaces between the bi-metal plate are filled with epoxy or other materials to prevent them from oxidizing and having degraded performance in humid environments. In trial runs, there did not appear to be a significant difference in exotherm detection between potted and unpotted TEMs. However, considering the potential for condensation across freezing and warming cycles, the decision was made to incorporate potted TEMs in future designs.

This simple DTA prototype was assembled in about an hour using a legacy Campbell Scientific Inc. (Logan, Utah, USA) CR23X datalogger and supplies on hand. Campbell Scientific’s ShortCut utility was used to program the logger according to the millivolt range of selected TEMs. Differential voltage measurements between two wires are commonly used for very fine scale or extremely low voltage sensing. This configuration allows for better noise rejection than single ended measurements which compare the voltage difference between a single wire and a ground. It was quite surprising to observe that the voltage from the TEMs selected for this prototype could be measured on single-ended channels without any noticeable decline in sensitivity. As a result, the number of TEMs that can be measured with a CR23X increases from 12 to 24.

This simple DTA prototype accommodates five sample wells made from adhesive foam insulation fitted with different TEMs covered with pieces of insulated bubble envelope material and two type T thermocouples connected to a CR23X datalogger. The purpose of the insulation was to create a well to hold the sample tissue and loosely insulate the tissue to ensure that exotherms that may be quite small, and ephemeral would be detectable by the TEM before they dissipate. Further, the cardboard enclosure that the prototype sits in serves to reduce air turbulence in the temperatue chamber so that exotherms are not stripped away before they can be detected. When the logger program starts, voltage and temperature measurements are made every second and the maximum, minimum and average values are recorded every 10 s. To collect exotherm data, plant tissue samples, such as dormant buds, are placed on top of the TEMs, covered with insulation, and placed in a programmable chamber. After a period of equilibration at ∼+4 °C, the programmable chamber slowly ramps down temperature (∼ −2 to −6 °C per hour) to some minimum temperature likely to cause cellular damage. Depending on the tissue or species, minimum temperatures of −40 to −60 °C are typically used. DTA is dependent on the process of intracellular water supercooling to some low temperature, followed by freezing, which releases heat as a consequence of the phase change from liquid to solid water. TEMs are highly effective at detecting heat fluxes which are indicated by an increase in millivolt output. This heat flux is rather ephemeral, lasting seconds in small samples and minutes in larger ones. The millivolt output is logged alongside temperature recorded with the thermocouples. By plotting voltage and temperature output over time, exotherms are visually identified as spikes or peaks and the temperature at which they occur is easily determined.

With access to programmable chambers and legacy dataloggers such as the CR23X, laboratory assessment of plant cold tolerance by means of DTA methodology can be achieved with DIY configurations at relatively low cost. There are pros and cons with using DTA compared to other techniques, such as relative electrolyte leakage (REL). Advantages include, minimal sample preparation, no chemicals or long incubations, and samples can be run overnight in the laboratory. However, it is somewhat limited by its dependence on the supercooling properties of individual species and tissue-types and may not be universally diagnostic. For example, cold tolerance of leaf tissue using DTA can be difficult to assess due to the lack of distinct exotherm peaks.

In summary, the DTA prototype and methodology can support research on plant cold tolerance by:

• Determining risk thresholds for winter injury of plant shoots or flower buds during vulnerable periods
• Informing decisions on deploying plant species and geographic provenances within species grown in seasonally cold environments
• Phenotyping forest and horticultural tree species in breeding programs to better define geographic planting limits
• Reducing labor and required handling times of tissue in laboratory cold tolerance assays compared to other techniques (e.g., REL)

## 3 Design files summary

The design of this device including components, electrical connections and wiring is presented entirely with photographs in section 5. Build instructions, and no CAD software was employed. The software code developed for both the CR23X and substitute logger CR1000X are available on the Zenodo repository (Table 1).

**Table 1:** List of data logger software code.

**Design file name**	**File type**	**Open source license**	**Location of the file**
TEM TEST SE.DEF, TEM TEST SE.DLD, TEM TEST SE.SCW	CR23X program files	Public Domain, Creative Commons Zero (CCO) Deed − CC0 1.0 Universal − Creative Commons	https://doi.org/10.5281/zenodo.17228047“CR23X program files.zip”
CR1000X TEM SE.CR1X, CR1000X TEM SE.DEF, CR1000X TEM SE.SCW,CR1000X TEM SE.TDF	CR1000X program files	Public Domain, Creative Commons Zero (CCO) Deed − CC0 1.0 Universal − Creative Commons	https://doi.org/10.5281/zenodo.17228047“CR1000X program files.zip”

The datalogger control software code for both loggers were generated with Campbell Scientific’s ShortCut programming utility (*.SCW file). TEM TEST SE.DLD needs to be uploaded to the CR23X logger to operate the DTA prototype and TEM TEST SE.DEF defines the data storage locations on an external computer.

## 4 Bill of materials summary

The construction of the DTA prototype requires a datalogger, software to connect to an external computer and generate code for operations, as well as the other electro-mechanical components. All of the components necessary to build and operate the DTA prototype, including their specifications and the suppliers from which each part was purchased are presented in Table 2.

**Table 2:** Bill of material for the simple DTA prototype.

**Designator**	**Component**	**Number**	**Cost per unit − USD**	**Total cost −** **USD**	**Source of materials**	**Material type**
Logger	Campbell Scientific 23X	1	0	0	Not commercially available	Data logger used from stock on hand
Logger*	Campbell Scientific CR1000X #33268–11	1	$2,031.23	$2,031.23	https://www.campbellsci.com/cr1000x	Substitute data logger
PC400 control software	Campbell ScientificPC400	1	0	0	Free download https://www.campbellsci.com/pc400	Control software
ShortCut programming utility	Campbell ScientificShortCut	1	0	0	Free download https://www.campbellsci.com/shortcut	Programing utility
Battery	Power-Sonic AGM General Purpose PS-1270 7Ah 12 V	1	$16.95	$16.95	https://www.batteryjunction.com/powersonic-ps-1270	Battery
Battery charger	Power-Sonic PSC-124000ACX 12 V	1	$49.95	$49.95	https://www.batteryjunction.com/powersonic-psc-124000acx	Battery charger
TEM TE-65P	TE-65–0.6–1.5; “potted”	1	$46.60	$46.60	https://tetech.com/peltier-thermoelectric-cooler-modules/micro/	Thermoelectric module
TEM TE-65	TE-65–0.6–1.5; “unpotted”	1	$40.90	$40.90	Same	Thermoelectric module
TEM TE-109P	TE-109–0.6–0.8;“potted”	1	$60.70	$60.70	Same	Thermoelectric module
TEM TE-109	TE-109–0.6–0.8;“unpotted”	1	$55.00	$55.00	Same	Thermoelectric module
TEM HP-127P	HP-127–1.4–1.5–72; “potted”	1	$33.80	$33.80	https://tetech.com/product/hp-127–1-4–1-5–72/	Thermoelectric module
Board	Prototype Board, MC01005, 1.6 mm, 160 mm, 115 mm	1	$6.09	$6.09	https://www.newark.com/multicomp-pro/mc01005/prototype-board-phenolic-160-x/dp/22AC8320?st = matrix%20board%20160 mm%20x%20115 mm	Mounting board
Wire	Tinned copper 22 AWG wire kit, 6 colors × 25′	1	$33.00	$33.00	https://www.rpelectronics.com/10-ht22k6-25.html	Wire
Thermocouple	T Type Thermocouple Extension Wire 25′	1	$45.11	$45.11	https://www.omega.com/en-us/temperature-measurement/temperature-wire-and-cable/thermocouple-wires/extt-tx-wire/p/EXTT-T-24–25	Thermocouple extension wire
Barrier Strip	Euro-style Barrier Strip 12 Position, 22–12 AWG, 20A, 300 V	1	$4.31	$4.31	https://www.rpelectronics.com/pa7ds.html	Wire connector
Foam tape	1–1/4 in. x 3/16 in. x 30 ft. Camper Mounting Tape	1	$6.93	$6.93	https://www.homedepot.com/p/Frost-King-1–1-4-in-x-3–16-in-x-30-ft-Camper-Mounting-Tape-for-Trucks-V447H/100122697	Insulation
Cover	A single bubble/paper mailer was cut to size to make TEM covers	1	$0.50	$0.50	https://www.uline.com/BL_1257/Uline-Self-Seal-Gold-Bubble-Mailers	Insulation
Enclosure	Cardboard box OD H x W x L	1	0	0	Stock on hand	Enclosure

## 5 Build instructions

**Fig. 1:**
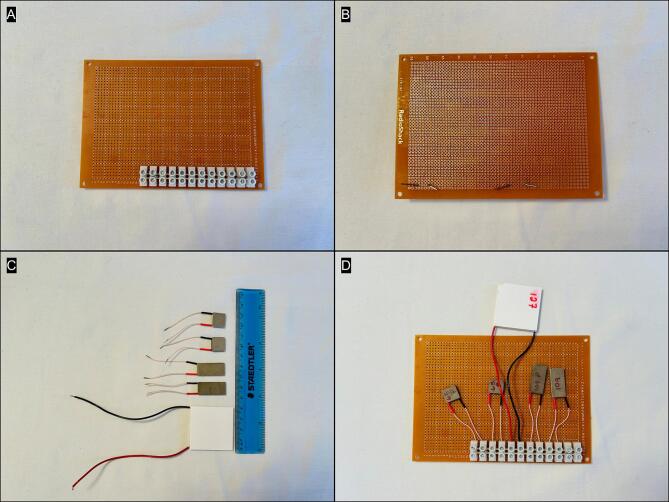
Layout of the prototype board showing the placement of the barrier strip (A, B), the relative size and dimensions of the TEMs (C), and connection of the TEMs to the barrier strip (D).

Attach barrier strip to prototype board (Fig. 1A). Paperclips were used to pin the barrier strip in place (Fig. 1B). More permanent construction would entail drilling holes through the prototype and securing the barrier strip with bolts and nuts. The individual TEMs are shown with a size reference in Fig. 1C. Connect TEMs to barrier strip with red (+) in first position and black (−) in second position for each. The TEMs were ordered as follows: TE-65P, TE-65, HP-127P, TE-109P, TE-109 (Fig. 1D).

**Fig. 2:**
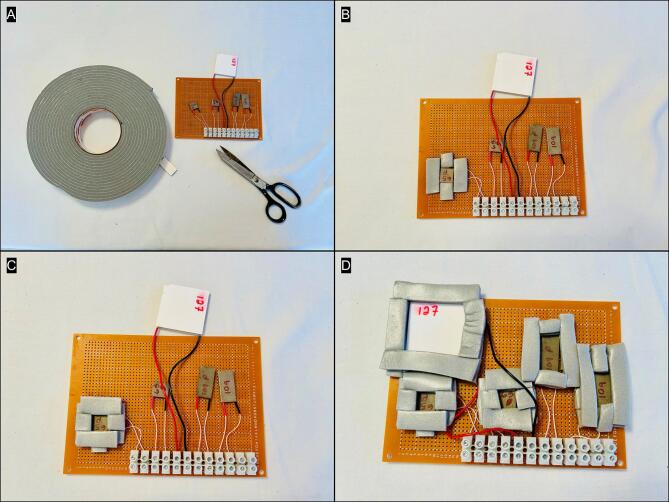
Assembly of foam wells around each TEM using adhesive foam weather stripping (A), that is applied in 2 overlapping layers (B, C) and completed for all five TEMs (D).

To create the wells that hold plant material on top of each TEM, first cut the adhesive weather stripping into strips ∼ 1 cm wide with scissors (Fig. 2A). Frame each TEM in two overlapping layers of adhesive weather stripping to create a well with sides slightly higher than the surface of the TEM (Fig. 2B-C). Repeat this process for the four remaining TEMs (Fig. 2D).

**Fig. 3:**
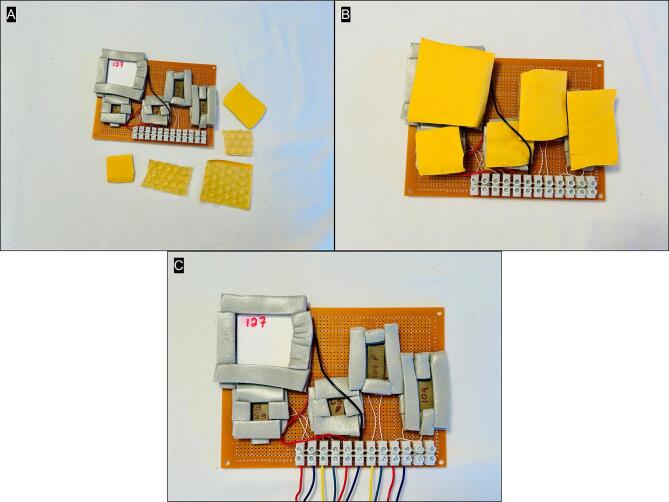
Padded bubble mail envelopes are cut to fit over the TEM wells (A, B), and extension wires are connected to the barrier strip to connect to with the datalogger, using either red or yellow for (+) terminal on TEM and green or blue for the (−) terminal (Fig. 3C). (For interpretation of the references to colour in this figure legend, the reader is referred to the web version of this article.)

Create insulated and protective covers for each TEM by cutting the padded bubble mail envelope to size (Fig. 3A). The covers should be sized to completely cover the TEM and the surrounding foam (Fig. 3B). Attach extension hook-up wires 2–3 m long to barrier strip to connect to with the datalogger (in later steps), using either red or yellow for (+) terminal on TEM and green or blue for the (−) terminal (Fig. 3C).

**Fig. 4:**
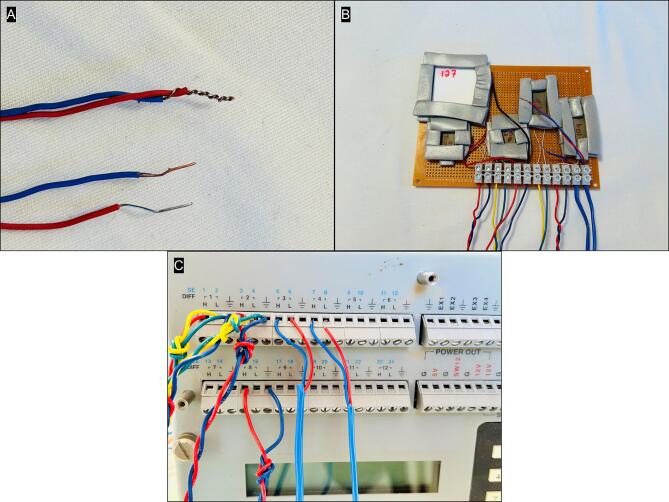
Steps related to making thermocouple junctions from extension wires (A), positioning the thermocouples at two locations over the prototype board (B) and wiring to connect the DTA prototype board to the Campbell CR23X datalogger (C).

Create a thermocouple junction by stripping one end of the thermocouple extension wire (total wire length 2 to 3 m), and twisting the bare wires tightly together. On the other end, simply strip the wire bare for later connection to the datalogger (Fig. 4A). Though not depicted in Fig. 4A, it is advisable to lightly solder the twisted thermocouple junction for best performance and reliability over time. Repeat steps to make thermocouple junction wire set. Another option is to install thermistors for even greater accuracy. Raise the screws on the barrier strip and insert the 2 thermocouples several cm in to sample ambient temperature in two locations, and gently tighten screws just enough to hold, but not damage the insulation (Fig. 4B). Connect wires to data logger as follows (Fig. 4C):

TEM1, red ∼ SE1, blue∼

TEM2, yellow ∼ SE2, green∼

TEM3, red ∼ SE3, blue∼

TEM4, yellow ∼ SE4, green∼

TEM5, red ∼ SE15, blue∼

Thermocouple 1, red ∼ SE5, blue ∼ SE6

Thermocouple 2, red ∼ SE7, blue ∼ SE8

**Fig. 5:**
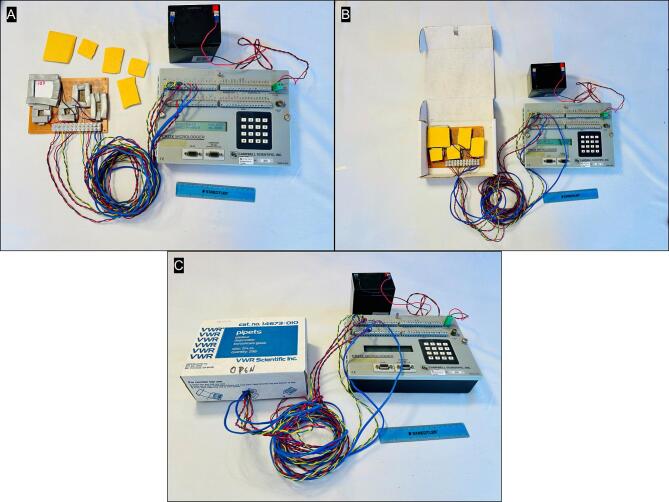
Final assembly of the DTA prototype with connection to a 12 VDC battery (A), and use of an enclosure, in this example a cardboard box (B & C) to limit air turbulence across the prototype board caused by fans in the temperature chamber.

Connect 12VDC battery to the logger via the green connector labeled with (+) and (−) (Fig. 5A). Enclose DTA prototype in cardboard box to minimize fan turbulence when placed in programable temperature chamber (Fig. 5B-C).

## 6 Operation instructions

These operation instructions are for a discontinued CR23X datalogger, which may be found on the secondary market for pre-owned scientific instruments. There are minor operational and programming differences with the substituted CR1000X logger listed in the Bill of materials summary section.

### 6.1 Software specifications

The Shortcut utility (Campbell Scientific, Inc., Logan, UT, USA), can be used to produce datalogger programs using a graphical user interface (GUI). There are three primary filetypes that are produced by the ShortCut utility: *.SCW which summarizes all options selected in the ShortCut GUI, *.DEF defines the physical wiring locations on the logger and names the data output labels, and *.DLD which is the compiled machine language that is sent to and used by the datalogger (Table 1). The prototype control program measures all TEMs every second, and logs maximum and mean millivolt values every 10 s. All TEMs were programmed for a single-ended measurement with a 50 mV range, measurement integration set to “Reject 60 Hz Noise (16.667 ms), multiplier = 1, offset = 0. Datalogger program files for the CR23X and the alternative CR1000X stored in the Zenodo repository [9].

### 6.2 Operation steps

1) Connect a computer to the CR23X datalogger using PC400 software (Campbell Scientific Inc., Logan, UT, USA) with a generic USB to serial cable, go to the “Clock/Program” tab, synchronize the datalogger to the computer’s clock and send the logger program named “TEM TEST SE.DLD”. This will start the program and measure voltages and temperature every second, record mean and maximum voltages every 10 s, and record average temperature every ten seconds.
2) Pre-cool the programmable chamber to + 4 °C.
3) Within the PC400 select the “Monitor Data” tab, select “Add” and add fields for voltage and thermocouple data. Test that the prototype is working by placing a finger on one or more TEMs and monitor the voltage response (+) which should spike after the rolling 10 s observation window turns over. If the voltage spike is negative, the single ended connections to the logger may be reversed or TEM needs to be turned over to the opposite side.
4) Depending on the size of the TEMs, place shoots (10 to 20 mm long) or buds on the TEMs. In the biological case example (Section 7), I placed buds directly on the TEMs and put sticky excised foliage in aluminum foil prior to placing on the TEM. Some researchers recommend placing plant tissue on moistened laboratory tissue and misting them with water to better differentiate high and low temperature exotherms [10].
5) Place bubble mailer insulation over each TEM, close cardboard box to reduce turbulence from chamber fans and place in programmable chamber.
6) Equilibrate and freeze plant tissue to pre-determined time and temperature. In this case, tissue was allowed to equilibrate for 12 h at + 4 °C then was ramped down at a rate of −4 °C per hour until −40 °C was reached 11 h later.
7) After the freezing cycle is complete connect to the CR23X logger with a computer, from the “Collect Data” table select “Collect new data from datalogger”, select “Table1″, click on button labeled “Start Data Collection” and verify that the data have been downloaded. The file format is *.DAT but is in comma separated value format and the extension can simply be changed to *.CSV.
8) Stop the data logger from continuously running by unplugging it.
9) After the chamber has returned to room temperature remove the plant tissue. If necessary, clean sticky residue with a laboratory tissue and isopropyl alcohol. Let any condensation dry from the chamber and TEM board.

#### 6.2.1 Operational notes

These instructions are specific to the red spruce bud biological case example in Section 7. Depending on the species, tissue type and state of dormancy; other tissue preparation, equilibration times, ramp rates and minimum temperatures will likely be necessary [1], [6], [7], [8], [11], [12].

### 6.3 Interpretation

The results may be visualized in a spreadsheet of choice or in R programming language. When the temperature is in a constant controlled rate of change, temperature can be displayed on the x axis and TEM voltage output on the y axis. In situations where the temperature is held steady for extended periods, visualization of TEM response on the y axis, time elapsed or observation number on the x-axis and chamber temperature on the secondary y axis may be more appropriate. The point where heat flux is first detected by the TEM is the temperature that induced the exotherm. Biological samples often have multiple or complex LTE peaks that make it difficult to automatically detect with software and separate from high temperature exotherms (HTEs) that may be generated by non-lethal intercellular freezing. Code was written in R to visualize data from each TEM in static graphs with the “ggplot2” package [13] and in a self-contained html interpretive tool using the “plotly” package [14]. The html file opens in a web browser allowing a user to pick exotherms and extract temperature data. Sample data, R code and output files (pdf and HTML) are in a compressed file (“interpretation.zip”) available on Zenodo [9].

#### 6.3.1 Html tool instructions and interpretation

**Fig. 6:**
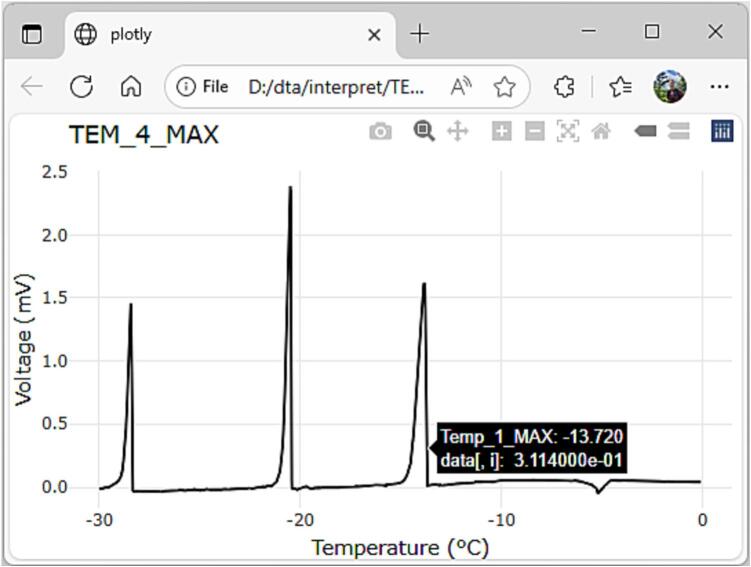
Screenshot of html interpretation tool used to identify three low temperature exotherms and manually extract temperature data. The first exotherm occurred at a temperature of −13.72 °C.

Extract the files from interpretation.zip, then run the R program “DTA interpretation.R” with the associated data file “sample_data_09022025.csv” with the proper working directory for your computer. The data file contains TEM response voltages for several saltwater solutions with different freezing points. The program will generate and save a pdf document with a plot of temperature on the x axis and TEM voltage on the y axis for each TEM (5) and separate self-contained html files for each TEM. The html files may be clicked on and opened in a computer’s default web browser. In the example in Fig. 3, Fig. 6, 3 different solutions were placed on the larger format HP-127P TEM yielding 3 exotherms. By dragging the cursor or pointer across the browser’s window a pop-up will show the temperature when the pointer crosses the plotted line. To manually pick an exotherm, drag the pointer across the screen from right to left and cross the plotted line where it first begins to spike above baseline (Fig. 6). In the example from TEM #4, exotherms were noted at −13.72°, −20.42° and −28.36 °C. The magnitude of the voltage spike is dependent on the properties of the TEM and the magnitude of the heat flux but is not typically used in the DTA assay.

## 7 Validation and characterization

The TEM’s are highly responsive to minute temperature differences and rapidly produce a millivolt signal when touched with a warm finger making it simple to test connections and ensure the polarity is the same across all units (i.e., temperature increase detected as a positive signal). Validation of measures across TEMs under freezing conditions is difficult to perform with dormant plant tissue due to variation between individual buds or other types of tissue, and once a bud is tested its cell walls are ruptured and cannot be used for another comparison. For this reason, I used several concentrations of sea salt diluted in deionized water to validate that freezing detection was consistent across TEMs, combined with a biological case example of red spruce buds with different cold hardiness levels to demonstrate the utility of the prototype.

### 7.1 Validation with sea salt dilutions

**Fig. 7:**
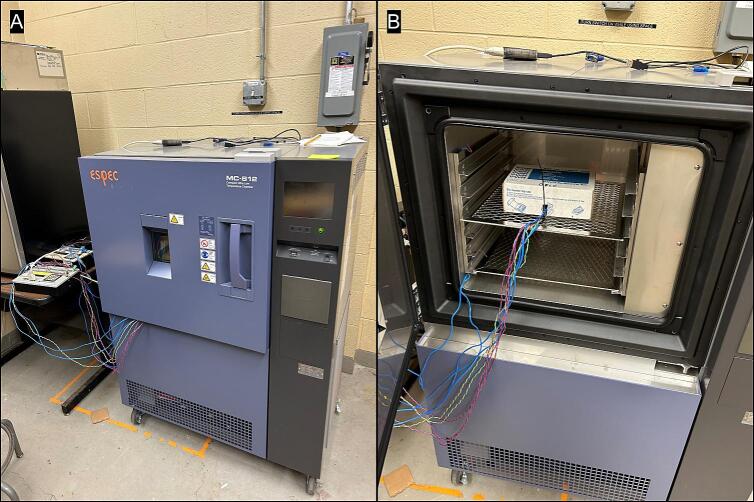
A) The programmable ultra-low temperature chamber (MC-812, ESPEC North America, Inc., 4141 Central Parkway, Hudsonville, MI 49426 USA) with prototype TEMs inside. The wires were thin enough to pass through the door opening. B) Another view of the programmable chamber with the door open.

To create standards with different freezing points and confirm that TEMs are operating similarly, four sea salt solutions were prepared. Twenty grams of sea salt were brought to a volume of 100 ml by adding deionized water while stirring. After the salt was completely dissolved, the 20% solution was used to make additional 5%, 10%, and 15% dilutions. For reference, mean ocean water salinity is ∼ 3.5% and the maximum amount of sea salt that can be dissolved in water is ∼ 23%. A variety of volumes and vessels were used to hold these solutions and were placed on the TEMs to evaluate whether they detected the release of the latent heat of fusion in a consistent manner. Initial attempts utilized a standard hole punch (6 mm diameter) from filter paper. These hole punches were placed on transparent adhesive tape, 5 µl of salt solution were placed on the paper and they were sealed closed with another piece of tape on top. The caps from 1.5 ml microcentrifuge tubes were removed and used as vessels containing 60 and 125 µl that were sealed with parafilm™. The samples were run in a series of tests using a freezer profile slightly different than that used in the biological case example (Section 6, operating instructions). A programmable ultra-low temperature chamber (MC-812, ESPEC North America, Inc., 4141 Central Parkway, Hudsonville, MI 49426 USA) was pre-cooled to + 3 °C (Figs. 7A-B). Once the temperature was steady, the chamber was programmed to soak (hold constant) at + 3 °C for 3 h, then ramp (controlled transition) temperatures down at a rate of −6 °C per hour and hold at −5 °C for 5 h, followed by another ramp to –32 °C at the same rate. The purpose of holding at 3 °C and −5 °C was to ensure thorough penetration of cold temperatures in the prototype materials.

Preliminary tests with the 6 mm punches moistened with 5 µl of salt solution indicated that the release of heat was highly detectable from each sample, but that they did not make adequate standards as there was evidence of evaporation over repeated measures and overlap in the exotherm detection between the 10% and 20% salt solutions.

#### 7.1.1 Validation test #1

Based on observations from the 6 mm punch assay and the possibility that the standards may have different exotherms specific to a given sample, the following plan was devised and implemented over 4 days. Four 60 µl salt solution samples (5%, 5%, 10%, 20%) were numbered 1, 2, 7, 3 (Table 3), measured on each of the smaller TEMs (TE-65, TE-65P, TE-109, TE-109P) over 4 consecutive days and the results were individually tracked. The larger TEM (HP-127P) could accommodate 3 dilutions (5%, 10%, 20%) numbered 4 through 6 (Table 3), so they were left in place for each day’s assay. The same ultra-low temperature chamber was used to perform the controlled temperature profile. The data were downloaded the following morning. The solution samples were moved to the next TEM and the process was repeated until each dilution was assayed on each of the 4 smaller TEMs (Table 4). The html interpretation tool was used to manually pick temperatures where exotherms were detected.

**Table 3:** Sea salt concentration in water for samples 1 through 7 for validation test #1.

Sample #	Concentration
S1	5%
S2	5%
S3	20%
S4	5%
S5	10%
S6	20%
S7	10%

**Table 4:** Experimental design of validation test #1 tracking the placement of sea salt in water samples (Table 3) on 5 TEMs across 4 assay days. The HP-127P TEM accommodated 3 samples at the same time due to its relatively large size.

TEM ID	Day 1	Day 2	Day 3	Day 4
TE-65	S3	S7	S1	S2
TE-65P	S7	S1	S2	S3
TE-109	S1	S2	S3	S7
TE-109P	S2	S3	S7	S1
HP-127P	S4	S4	S4	S4
HP-127P	S5	S5	S5	S5
HP-127P	S6	S6	S6	S6

##### 7.1.1.1 Validation test #1 results and discussion

Samples 1, 2, 3, and 7 were rotated across the four smaller TEMs over four days yielding consistent exotherm detections as evidenced by standard errors ranging from 0.08 °C to 0.28 °C (Table 5). Despite best attempts to create standard salt solutions, there were notable differences in freezing point between sample 1 and 2 even though they contained the same concentration of salt. This difference was inherent in the samples and was observed regardless of TEM (Table 5). Samples 4, 5, and 6 were left in place on HP-127P for all four measurement days. Samples 5 and 6 yielded consistent exotherm detection with standard errors of 0.13 °C to 0.36 °C over the four days. The freezing temperature of sample 4 decreased by more than 4 °C over the 4-day experiment. These results highlight both the consistency of using TEMs for exotherm detection and the challenges of trying to create a standard with a fixed, repeatable freezing point. I would contend that the TEMs paired with the datalogger are very effective at detecting exotherms and most of the variation originates with the “standard”. Creating solutions that freeze at specific temperatures can be challenging since supercooling and subsequent ice nucleation is dependent on salinity, pressure, contaminants, droplet size and velocity of cooling [15], [16], [17] and crystallization may also be induced by vibration or contact with the wall of a vessel [18]. The TEMs are very effective at detecting heat flux, though the standard solutions have numerous factors that can affect their freezing properties and points. Contaminants such as a piece of dust or fiber, or uneven contact with the vessel wall, could have caused sample 2 to crystallize at a warmer temperature than sample 1, though it remained consistent throughout the experiment. The gradual decline in freezing point for sample 4 could represent leakage or a change in vessel wall contact as the prototype was handled each day. Despite some issues with creating ice nucleation or freezing point standards, there was evidence of consistent performance across TEMs.

**Table 5:** Results of validation test #1 showing the temperature (°C) where exotherms were detected sorted by sample number across all TEMs.

Sample #	S1	S2	S3	S4	S5	S6	S7
Salt Concentration	5%	5%	20%	5%	10%	20%	10%
	−15.84	−10.38	−28.34	−13.74	−20.42	−28.37	−18.55
	−16.22	−10.08	−28.25	−14.16	−19.72	−28.68	−19.48
	−15.98	−10.2	−28.6	−17.55	−19.14	−28.34	−19.28
	−15.92	−10.43	−28.83	−18.08	−18.79	−28.87	−19.89
Summary Statistics							
Mean	−15.99	−10.27	−28.51	−15.88	−19.52	−28.57	−19.30
Std. Deviation	0.16	0.16	0.26	2.25	0.71	0.25	0.56
Std. Error	0.08	0.08	0.13	1.12	0.36	0.13	0.28

#### 7.1.2 Validation test #2

A second test was conducted to address issues related to the freezing standards in validation test #1. The vessels were filled with 125 µl of salt solution where all wall surfaces were evenly in contact with the solution and a concave water surface was observed. Salt solutions freeze in two phases. The first exotherm is generated when pure water freezes into a lattice and salt is largely excluded but remains in a brine [19], [20]. As cooling continues, the concentrated brine reaches its eutectic temperature and the remaining liquid solidifies as a mixture of ice, pure salt crystals and salt hydrates thereby creating a second exotherm [19]. In test #2 the minimum temperature was lowered to −41 °C to detect the second exotherm at the eutectic temperature. Regardless of the salt concentration of each solution and temperature of the first exotherm, they should all share a common, detectable second exotherm at the eutectic point. On four consecutive days salt solutions of 5% (day 1), 10% (day 2), 15% (day 3), 20% (day 4) were placed on each of the 5 TEMs in the prototype. The same ultra-low temperature chamber from validation test #1 was used and the temperature profile was re-programmed with a minimum temperature of −41 °C. The data were downloaded the following morning and the assay repeated for the remaining dilutions. The html interpretation tool was used to manually pick temperatures where first and second exotherms were detected.

##### 7.1.2.1 Validation test #2 results and discussion

**Fig. 8:**
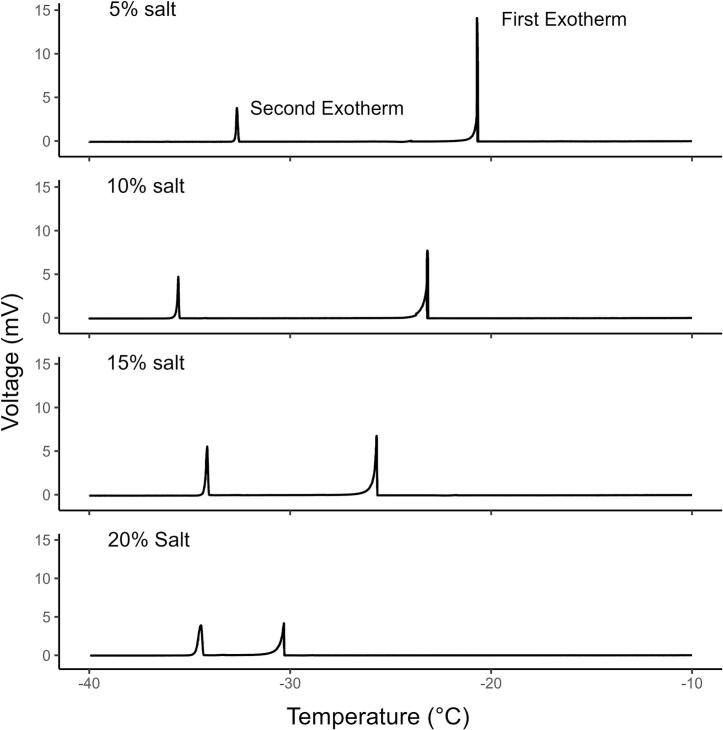
Sample exotherm detection from the same thermoelectric module (model TE-65P) for four different sea salt dilutions measured over four consecutive days. The first exotherm occurs at warmer temperatures and indicates initial freezing and exclusion of salt brine. The second exotherm is detected at the eutectic temperature where remaining brine is converted into a mixture of ice, pure salt crystals and salt hydrates.

Lowering the chamber temperature profile to −41 °C permitted the detection of 2 exotherms from each salt solution sample (Fig. 8). Solutions with lower concentrations of salt exhibited the first exotherm at warmer temperatures than solutions with high concentrations of salt (Fig. 8, Table 6). Across the different solutions the mean temperature of the first exotherm ranged from −19.16 °C to −29.75 °C, while the second exotherm mean temperature ranged between –33.14 °C and −34.82 °C (Table 6). As the salt concentrations increased from 5% to 20% there was a reduction in variance in both the first and second exotherm mean temperatures. Based on this evidence, the 20% salt solution may exhibit a more predictable, repeatable freezing point and serve as a better standard for future performance comparisons. Considering the more consistent nature of the second exotherm at the eutectic temperature, the results of test #2 were also compared by TEM model (Table 7). With the exception of TE-65, the mean temperature of the second exotherm was within 0.71 °C for the other 4 TEMs. It is doubtful that there is any substantive performance difference between the TEMs and the difference may be due to the spatial distribution within or specific location of TEMs in the cardboard enclosure. The TE-65 TEM was located near the barrier strip at center edge of the prototype board (Fig. 4). This is closer to the opening in the enclosure where the wires pass through into the temperature chamber than the other units (Fig. 5) and could result in more rapid cooling. The simplest solution may be better sealing of the opening or adding a temperature sensor to each TEM well in future embodiments. Considering there was no replication in TEM models or the environmental sealing options (potting indicated with a “P”) these were not performance tests between TEMs, but a validation of the proof of concept of the prototype. This validation test shows that they all are capable of detecting and quantifying the release of the latent heat of fusion which is known to occur in 2 stages in salt water [19], [20].

**Table 6:** Mean temperatures (°C) where the first and second exotherms were detected by salt concentration in validation test # 2.

Salt Concentration (%)	First Exotherm	s.e.		Second Exotherm	s.e.
5	−19.16	1.52		−34.82	1.03
10	−21.46	1.33		−34.31	0.46
15	–23.92	1.45		–33.62	0.52
20	−29.75	0.35		–33.14	0.24

**Table 7:** Mean temperatures (°C) where the second exotherms were detected across all four salt solutions (5%, 10%, 15%, 20%) by TEM model in validation test # 2.

Model	Second Exotherm	s.e.
TE-65	–32.57	0.10
TE-65P	−34.52	1.20
TE-109	−34.10	0.53
TE-109P	−34.25	0.52
HP-127P	−34.81	0.38

### 7.2 Biological case example

Dormant buds of red spruce (*Picea rubens* Sarg.) were used to demonstrate the ability of the DTA prototype to detect exotherms produced by plant tissue at specific temperatures. On May 19, 2024, dormant red spruce buds were collected from 2 locations (44.17955, −72.95922, 549 m elevation, and 44.16900, and −72.92958, 1,067 m elevation) on Mount Ellen in northern Vermont, and at the University of Vermont (UVM) Horticultural Education and Research Center in South Burlington, Vermont (44.43096, −73.20503, 69 m elevation). Foliage samples from 549 m and 1,067 m were also collected to utilize the remaining TEM wells. The samples were stored in a refrigerator until running the assay the next day. The rationale of sampling trees at different elevations was to demonstrate that trees were becoming less cold tolerant in response to warming springtime ambient temperatures at each elevation. Accordingly, samples collected from a lower, warmer elevation would be expected to be less cold tolerant and produce exotherms at higher temperatures upon incremental freezing. Likewise, samples collected from a colder high elevation location would be expected to be more cold tolerant and exhibit exotherms at comparatively lower temperatures with controlled freezing.

On May 20^,^ 2024, buds were trimmed from the stems and 3 buds from each elevation were placed in separate wells (TE-65, TE-65P, TE-109). The resinous foliage was wrapped in aluminum foil to protect the TEMs and placed on the TE-109P and HP-127P. Five different TEM models were used in the prototype and no quantitative assessment of how they might differ from each other was performed since all appeared to detect exotherms adequately and there was no replication by TEM model. Each TEM was covered with a rectangle of bubble insulation and the cardboard enclosure box was carefully closed and placed in the programmable ultra-low temperature chamber. The chamber was pre-cooled to + 4 °C (Figs. 7A-B). Once the temperature was steady, the chamber was programmed to soak at + 4 °C for 12 h and then ramp temperatures down at a rate of −4 °C per hour until −40 °C was reached. The data were downloaded the following day and the html interpretation tool was used to select exotherm temperatures. All data, R code and output files (pdf and HTML) are in a compressed file (“red_spruce_example.zip”) available on Zenodo [9].

**Fig. 9:**
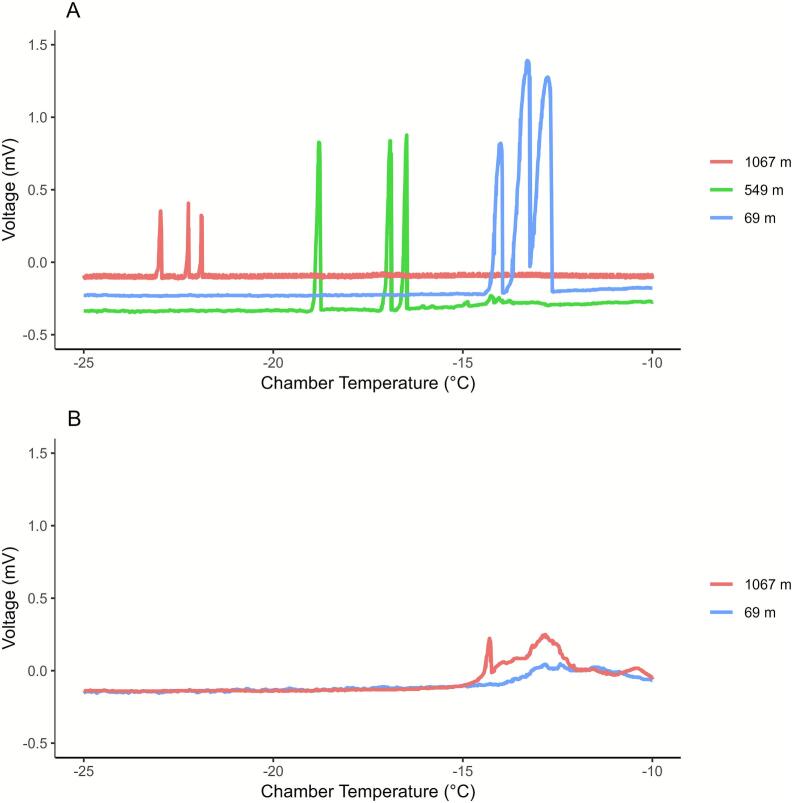
Air temperature in the programmable chamber and the TEM response to red spruce buds (A) and foliage (B) releasing the latent heat of fusion from supercooled intracellular water. Buds were collected at elevations of 69, 549, and 1067 m and placed directly on the TEMs. Foliage from 69 and 1067 m was wrapped in aluminum foil and placed on TEMs for analysis. (For interpretation of the references to colour in this figure legend, the reader is referred to the web version of this article.)

Since the chamber temperature ramped down at a controlled rate it was possible to plot the voltage produced by each TEM versus thermocouple temperature within the enclosure (Fig. 9). The buds produced clear low temperature exotherms (LTEs) when the supercooled intracellular water froze and the TEMs detected the release of the latent heat of water produced by the phase change. The TEMs were sensitive enough to individually detect the LTE of each bud (Fig. 9). The peak of each bud LTE was as follows: 69 m elevation −12.68, −13.23, −13.96 °C; 549 m elevation −16.48, −16.98, −18.74 °C; and 1067 m elevation −21.85, –22.22, –22.94 °C. The resulting exotherms indicate that red spruce buds are becoming less cold tolerant with decreasing elevation and increasing ambient temperatures. This de-acclimation phenomenon is well known and has been characterized using DTA for a variety of wild and cultivated woody species that experience strong seasonal variations in temperature [8], [10], [12], [21], [22]. The millivolt signal was much lower in the red spruce assay (∼0.2 to 1.5 mV) than observed with 125 µl salt water solutions but was still easily interpreted (Fig. 8, Fig. 9). The foliage samples did not produce crisp LTE peaks that could be quantitatively compared, both initiated broad exotherms at ∼ -9.6 °C. This was not unexpected and highlights the tissue specificity of DTA assays where supercooling of intracellular water and subsequent freezing is necessary for detection [23]. Species and tissues that use other avenues of protection such as dehydration may be better assayed using relative electrolyte leakage approaches [24]. Under mid-winter conditions many dormant woody plants produce HTEs in the range of −5 °C to −10 °C followed by LTEs at colder temperatures. This phenomenon results from non-lethal extracellular freezing and this was not observed in this assay with red spruce. This case example demonstrates the ability to use this simple DTA prototype for the detection of LTEs in a suitable species and tissue type, as well as the staggered de-acclimation of red spruce buds along an elevational gradient in the springtime.

### 7.3 Cost considerations and open-source substitutions

#### 7.3.1 Substitution of Campbell Scientific data logger with less expensive microcontrollers or single-board computers

This simple prototype is a proof of concept that describes how TEMs can be used to assess plant tissue cold hardiness with DTA. I used the hardware that I was familiar with and had access to demonstrate how TEMs can be repurposed as heat flux sensors to detect the release of the latent heat of fusion when water in plant tissue freezes. In the fields of plant physiology and environmental monitoring, Campbell Scientific Inc. dataloggers are widely used and available, but newer, more modern versions can be costly, thereby limiting their accessibility. In Section 4, a commercially available CR1000X is offered as a substitute if a legacy CR23X cannot be sourced. Less expensive voltage measurement systems such as the Arduino microcontroller [25], and the Raspberry Pi single-board computer [26] coupled with a high-resolution external Analog-to-Digital Converter (ADC) should be configurable to this prototype as long as they can measure voltage in the range of −20 to + 20 millivolts with a resolution of 0.1 millivolts, accept voltage output from a thermistor or thermocouple, be able make measurements every 5 to 10 s, and store internally or output up to 24 h of data to an external computer. These substitutions are offered as examples but have not been evaluated in the development of this DTA prototype.

#### 7.3.2 Substitution of commercial programmable temperature chamber with lower cost alternatives

To operate effectively, DTA systems require a freezer to expose plant tissue to temperatures ranging from + 4 °C to at least −45 °C depending on the species. Systems that gradually lower the chamber temperature at a precise rate like the ESPEC model MC-812 used in this study (Fig. 7) can be quite expensive and represent a significant cost (∼$10,000 + USD) outside of the DTA prototype. For plant breeding or genotype deployment guidelines, coarser temperature steps such as 2 to 5 °C [27], [28] followed by an equilibration period for 30 to 60 min at each temperature step may be appropriate for some applications and mitigate the need for a precision temperature chamber. While precision chambers may be available as a shared resource at universities or agricultural laboratories, I wanted to offer some lower cost alternatives to allow greater access to the DTA methodology. I have not evaluated their efficacy, but they seem likely to work based on precedence in the literature.

##### 7.3.2.1 Modification of chest freezers

Use of a less expensive chest freezer with a Arduino-based temperature controller [29] or step-cooling with a chest freezer equipped with a digital temperature readout that is set manually could serve as a viable alternative depending on the goals of the assay. Consumer grade chest freezers capable of −40 °C are available for $500 to $700 USD and commercial chest freezers capable of −45 °C available for $1,200 + USD.

##### 7.3.2.2 Passive cooling augmented with active heating

Both liquid nitrogen, dry ice and ethanol-dry ice mixtures [30] offer opportunities to lower temperature chambers down to a minimum temperature using passive cooling [31] and then achieve more precise temperature control with active heating. The concept is relatively simple where a chamber is made from an insulated box or cooler and has its headspace linked to a vessel containing liquid nitrogen, dry ice or, a combination of ethanol and dry ice. The headspace in the cooler will come into equilibrium at some low temperature based on the thermal properties of the headspace volume, insulative properties and amount of passive cooling substrate. The chamber is equipped with a fan to achieve temperature consistency and a thermocouple to monitor headspace temperature. Temperature control is then achieved with a heater linked to the thermocouple with a microcontroller to precisely set the temperature in the chamber [29]. The heater could be a electrical resistance type heater or a Pelletier device [32]. The headspace cooling approach may also be altered to a box within a box approach where an insulated box is directly placed in a bath of ethanol and dry ice (−78 °C) [30] and active heating is used for fine temperature control [32].

## Declaration of competing interest

The author declare that they have no known competing financial interests or personal relationships that could have appeared to influence the work reported in this paper.
